# Oral lesions in patients with systemic diseases: A hospital-based observational study

**DOI:** 10.6026/973206300214359

**Published:** 2025-12-15

**Authors:** Dharmendra Kumar M.G, Vijay Shekhar, Samir Mansuri, Shiny Joseph, Libni D. Angel, Jagadish Prasad Rajguru, Heena Dixit

**Affiliations:** 1 Department of Oral and Maxillofacial Surgery, C.K.S. Theja Dental College, Renigunta Road, Tirupati andhra Pradesh, India; 2 Department of Dentistry, Nalanda Medical College Hospital, Agamkuan, Patna, Bihar, India; 3 Consultant Oral and Maxillofacial Surgeon, Ahmedabad, Gujarat, India; 4 Department of Periodontics, Al Azhar Dental College, Thodupuzha, Kerala, India; 5 Department of Pathology, Sree Balaji Medical College and Hospital, Chennai, Tamil Nadu, India; 6 Department of Oral and Maxillofacial Pathology, Hi-Tech Dental College & Hospital, Bhubaneswar, Odisha, India; 7 Department of Medical Health Administration, Index Institute, Malwanchal University, Index City, Nemawar Road, Indore, Madhya Pradesh, India

**Keywords:** Oral manifestations, systemic diseases, oral lesions, hospital-based study, observational study

## Abstract

Systemic diseases frequently manifest through characteristic oral lesions, making the oral cavity an important indicator of underlying medical conditions. Therefore, it is of interest to assess the prevalence and patterns of oral lesions among patients with confirmed systemic diseases in a hospital-based population. Hence, a cross-sectional observational analysis of 500 adults was conducted, documenting demographic data, systemic diagnoses and clinically verified oral findings, which were evaluated using WHO criteria and analyzed statistically. The results demonstrated high frequencies of periodontal disease, candidiasis, glossitis, aphthous ulcers and lichenoid reactions, with distinct associations observed between specific lesions and conditions such as diabetes mellitus, hypertension, anemia, gastrointestinal disorders, autoimmune diseases and chronic kidney disease. These findings highlight the essential role of routine oral examination in the early detection and multidisciplinary management of systemic diseases.

## Background:

The oral cavity is often described as a window to overall health, since systemic conditions frequently manifest through oral changes [1]. Disorders such as diabetes mellitus, hypertension, anemia, gastrointestinal diseases, autoimmune disorders and chronic kidney disease are known to produce characteristic oral signs that can serve as diagnostic aids [2, 3]. Diabetes is strongly associated with periodontal disease, candidiasis and xerostomia [7, 8], whereas anemia presents with glossitis, cheilitis and pallor [11]. Gastrointestinal disturbances are often accompanied by recurrent aphthous ulcers and nutritional glossitis [5], while autoimmune diseases may lead to lichen planus and xerostomia [6, 9]. Chronic kidney disease manifests with stomatitis, candidiasis and altered mucosal appearance due to uremia and immune dysfunction [4, 13]. Oral physicians and dentists are frequently the first to detect such changes during clinical examinations, making them vital partners in multidisciplinary care [10]. Despite global studies reporting oral lesions in systemic conditions [12, 14 and 15], comprehensive hospital-based data from Indian populations remains limited. Therefore, it is of interest to assess the frequency, distribution and associations of oral lesions with systemic diseases in a hospital-based population, with an emphasis on diagnostic and preventive implications.

## Materials and Methods:

This cross-sectional observational study was conducted in the Department of Oral Medicine and Radiology of a tertiary care hospital over 12 months. Adults aged 18 years and above with confirmed systemic diseases were included. Patients unwilling to participate, with recent maxillofacial surgery, or on long-term corticosteroid therapy were excluded. A total of 500 participants were enrolled. Data on demographics, systemic disease history and oral health findings were recorded using a structured proforma. Oral examinations were performed under proper illumination using sterile instruments and lesions were categorized according to WHO oral health assessment criteria. Statistical analysis was conducted using SPSS version 25, with descriptive statistics and chi-square tests applied. A p-value <0.05 was considered statistically significant.

## Results:

The study population was predominantly middle-aged, with a male predominance and a larger proportion from urban areas. Among systemic diseases, diabetes mellitus (30%) and hypertension (24%) were the most common, followed by anemia and gastrointestinal conditions. Autoimmune disorders and chronic kidney disease were less frequent but still significant Table 1 (see PDF). Periodontal disease emerged as the most common oral lesion (24%), particularly among patients with diabetes mellitus, reflecting the strong bidirectional link between diabetes and periodontal health. Candidiasis was the second most prevalent lesion (18%), occurring in diabetes, anemia, autoimmune conditions and chronic kidney disease. Xerostomia was predominantly reported in autoimmune disorders and diabetes. Anemia patients showed a clear pattern of atrophic glossitis and angular cheilitis, while gastrointestinal conditions were strongly associated with recurrent aphthous ulcers. Hypertensive patients frequently presented with pigmentation and lichenoid reactions, likely linked to antihypertensive medications. Autoimmune conditions contributed to a higher incidence of lichen planus and xerostomia. Chronic kidney disease patients exhibited candidiasis and uremic stomatitis as distinctive findings Table 2 (see PDF).

## Discussion:

The present study demonstrated a high prevalence of oral lesions in patients with systemic diseases, emphasizing the diagnostic and prognostic value of oral examinations in hospital settings. Periodontal disease and candidiasis were the leading oral findings, consistent with earlier work linking diabetes mellitus to periodontal destruction and fungal infections [7, 8]. The strong association between diabetes mellitus and periodontal disease supports existing evidence of the two-way relationship between metabolic control and periodontal health [1, 2]. Hyperglycemia impairs host defense mechanisms, thereby predisposing individuals to infections such as candidiasis [9], while also promoting periodontal breakdown [7]. Hypertension was frequently associated with pigmentation and lichenoid lesions, likely attributable to long-term drug therapy [6]. These drug-induced oral manifestations pose diagnostic challenges but can be managed through careful clinical recognition. Anemia was strongly correlated with atrophic glossitis and angular cheilitis, which are classical indicators of nutritional deficiency and hematological imbalance [11]. These oral changes serve as early, non-invasive diagnostic markers that may direct patients toward appropriate hematological evaluation [5]. Gastrointestinal disorders were particularly associated with aphthous ulcers and glossitis, underscoring the impact of malabsorption and nutritional deficiencies [5, 12]. Autoimmune conditions revealed significant associations with lichen planus and xerostomia, highlighting the role of oral examination in identifying systemic autoimmune pathology [6, 9]. Chronic kidney disease presented with candidiasis and uremic stomatitis, reflecting both immunosuppression and uremic toxin effects [4, 13]. Overall, the findings confirm the crucial role of oral physicians and dentists in systemic disease detection and monitoring. Routine oral examination should be integrated into systemic disease management protocols, thereby promoting early diagnosis, timely referral and holistic patient care [10, 14 and 15].

## Conclusion:

Oral lesions are highly prevalent among patients with systemic diseases is shown. Periodontal disease, candidiasis, glossitis, aphthous ulcers and lichen planus were the most commonly observed conditions, with distinct associations noted for specific systemic disorders. Oral health evaluation should be recognized as an integral component of systemic disease management, necessitating close collaboration between medical and dental professionals.
